# Network Analysis Identifies Sex-Specific Gene Expression Changes in Blood of Amyotrophic Lateral Sclerosis Patients

**DOI:** 10.3390/ijms22137150

**Published:** 2021-07-01

**Authors:** Jose A. Santiago, James P. Quinn, Judith A. Potashkin

**Affiliations:** 1 NeuroHub Analytics, LLC, Chicago, IL 60605, USA; jose.santiago.ecm@gmail.com; 2 Q Regulating Systems, LLC, Gurnee, IL 60031, USA; jim.quinn@qregulatingsystems.com; 3 Center for Neurodegenerative Diseases and Therapeutics, Cellular and Molecular Pharmacology Department, The Chicago Medical School, Rosalind Franklin University of Medicine and Science, North Chicago, IL 60064, USA

**Keywords:** ALS, blood, co-expression networks, neurodegeneration, switch genes, sex differences

## Abstract

Understanding the molecular mechanisms underlying the pathogenesis of amyotrophic lateral sclerosis (ALS), a devastating neurodegenerative disease, is a major challenge. We used co-expression networks implemented by the SWitch Miner software to identify switch genes associated with drastic transcriptomic changes in the blood of ALS patients. Functional analyses revealed that switch genes were enriched in pathways related to the cell cycle, hepatitis C, and small cell lung cancer. Analysis of switch genes by sex revealed that switch genes from males were associated with metabolic pathways, including PI3K-AKT, sphingolipid, carbon metabolism, FOXO, and AMPK signaling. In contrast, female switch genes related to infectious diseases, inflammation, apoptosis, and atherosclerosis. Furthermore, eight switch genes showed sex-specific gene expression patterns. Collectively, we identified essential genes and pathways that may explain sex differences observed in ALS. Future studies investigating the potential role of these genes in driving disease disparities between males and females with ALS are warranted.

## 1. Introduction

Amyotrophic lateral sclerosis (ALS), also known as Lou Gehrig’s disease, is a devastating neuromuscular disease characterized by the progressive degeneration of motor neurons in the brain and spinal cord. Unfortunately, there is no cure for the disease, and patients frequently die from respiratory paralysis within two to five years after disease onset [1]. Genetic causes account for less than 10% of the cases. Most of the hereditary cases display an autosomal dominant pattern in genes encoding Cu/Zn superoxide dismutase (SOD1), TAR-DNA binding protein 43 (TDP-43, C9ORF72, and fused in sarcoma (FUS)) [2]. Other rare mutations in optineurin (OPN), valosin-containing protein (VCP), and ubiquilin 2 (UBQLN2) have been reported in familial cases [2]. Nevertheless, the vast majority of the cases are considered sporadic, suggesting that a complex interaction between genetics and environmental insults is responsible for the disease pathogenesis.

The major neuropathological features of ALS include the extensive degeneration of lower motor neurons from the anterior horn of the spinal cord and brainstem, the loss of Betz cells in the primary motor cortex, and degeneration of the lateral corticospinal tracts [1]. The different involvement in the upper and lower neuronal motor systems contributes to the marked phenotypic heterogeneity in ALS. Nuclear translocation and aggregation of TDP-43 is a pathological hallmark in most ALS cases.

Numerous risk factors have been associated with the development of ALS. Evidence from epidemiological studies indicates that older age, family history, and sex are established risk factors for the disease [3]. Like other neurodegenerative diseases, sex-specific differences have been reported in animal models and clinical studies [4,5]. Epidemiological studies from different populations have consistently shown that males are at higher risk for the disease than females [6,7]. The mechanisms underlying sex-specific differences, however, remain poorly understood. In addition to sex and older age, other risk factors, including type 2 diabetes, dietary factors, body mass index, physical fitness, and exposure to chemicals, have been implicated in the pathogenesis of ALS [3].

Clinical trials testing potential drugs for ALS have been partly unsuccessful due to the lack of sensitive and specific biomarkers. Growing interest in precision medicine is fueling research to identify biomarkers for identifying presymptomatic ALS patients. For example, levels of neurofilament light (NfL) were higher in CSF and serum of ALS and presymptomatic patients compared to healthy controls [8]. Changes in expression of NfL were observed 6–12 months before the manifestation of the earliest clinical symptoms. Nevertheless, NfL levels have been shown to predict neurodegeneration and progression in presymptomatic AD patients thereby suggesting it may not be a specific biomarker for ALS [9]. In addition, the phosphorylated neurofilament heavy subunit (pNFh) was increased in blood and CSF of ALS patients and associated with disease progression [10]. Inflammatory markers IL2, IL5, IL6, and IL8 were differentially expressed in the blood of ALS patients compared to controls [11]. Despite this progress, a fully validated prognostic and diagnostic biomarker is yet to be discovered.

Transcriptomic and network-based analyses have been instrumental in identifying biomarkers, therapeutic targets, and disease mechanisms in neurodegenerative diseases [12,13]. One successful approach is the analysis of co-expression networks to identify “switch genes”; genes responsible for drastic gene expression changes that may play a fundamental role in disease pathogenesis. The SWIM software has been developed for facilitating the identification of switch genes. This network analysis software has been used successfully to identify key switch genes and biological pathways in Alzheimer’s disease, vascular dementia, and frontotemporal dementia [14,15] and glioblastomas [16]. This is the first study to employ SWIM in datasets from ALS patients to unveil genes and biological pathways that may play a fundamental role in the pathogenesis of ALS. The sex differences identified in this study may provide valuable information to scientists and clinicians working towards developing better personalized treatments for ALS patients.

## 2. Results

### 2.1. Database Mining for ALS Transcriptomic Studies

**Figure 1:**
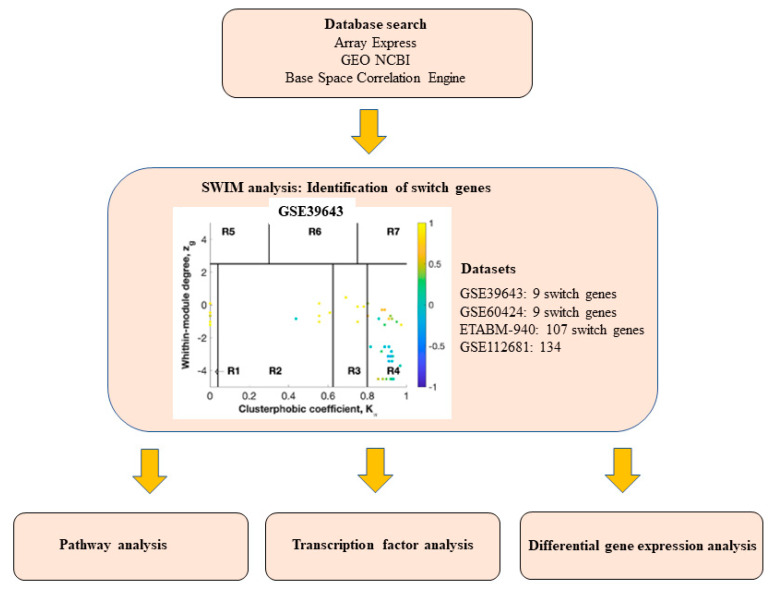
Overall study design. Array Express, NCBI GEO, and BSCE databases were searched for human transcriptomic studies in ALS. Microarrays were analyzed separately using the SWIM algorithm to identify switch genes. Biological and functional analyses of switch genes were performed using NetworkAnalyst and miRNet.

A flow chart describing the overall strategy we used to identify switch genes and analyze networks is presented in Figure 1. We interrogated the Array Express, NCBI GEO, and Base Space Correlation Engine databases to identify gene expression studies from ALS patients and age-matched controls. We focused our analysis on blood studies containing human samples from sporadic ALS patients. Seven arrays containing gene expression data from ALS patients and age-matched healthy controls were identified and considered for further analysis (Table 1).

### 2.2. Identification of Switch Genes in ALS

We performed the SWIM algorithm on the microarrays described in Table 1 in order to unveil key switch genes that may be associated with the pathogenesis of ALS patients, as described previously [14,15,16]. Gene expression datasets were imported into SWIM separately. Four microarrays GSE60424, GSE39643, E-TABM-940, and GSE112681, containing blood transcriptomic data from ALS patients, were robust enough to identify switch genes after adjusting the required fold change and *p*-value. These arrays were considered for further analysis.

**Figure 2:**
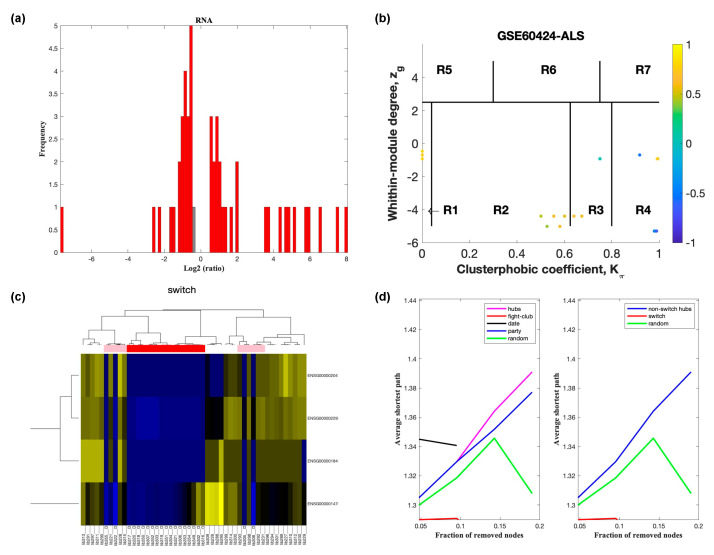
SWIM analysis of whole blood of ALS subjects in GSE60424. (**a**) Distribution of log2 fold change values where the red bars are selected for further analysis. (**b**) Heat Cartography Map with nodes colored by their average Pearson Correlation Coefficient. Region R4 represents the switch genes. (**c**) Dendrogram and heat map for switch genes. The suffix ____D indicates the sample came from the diseased cohort. (**d**) Robustness of the correlation network.

**Figure 3:**
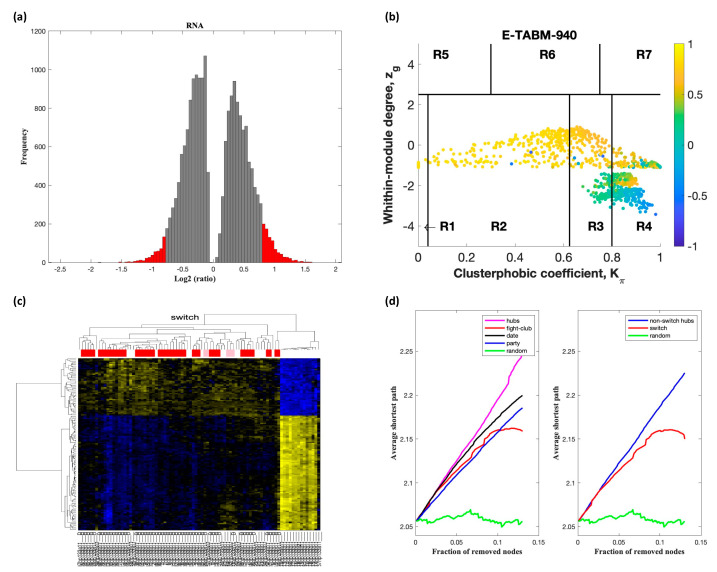
SWIM analysis of whole blood of ALS subjects in E-TABM-940. (**a**) Distribution of log2 fold change values where the red bars are selected for further analysis. (**b**) Heat Cartography Map with nodes colored by their average Pearson Correlation Coefficient. Region R4 represents the switch genes. (**c**) Dendrogram and heat map for switch genes. The suffix ____D indicates the sample came from the diseased cohort. (**d**) Robustness of the correlation network.

**Figure 4:**
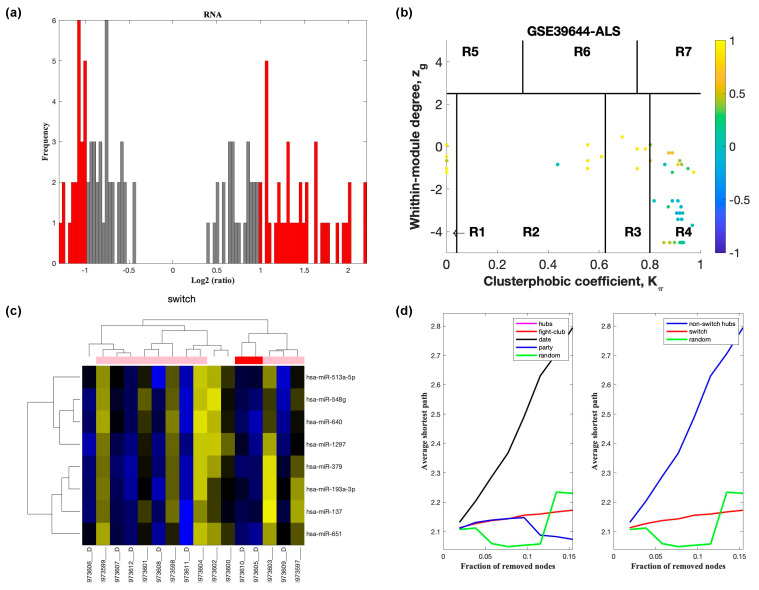
SWIM analysis of monocytes of ALS subjects in GSE39643. (**a**) Distribution of log2 fold change values where the red bars are selected for further analysis. (**b**) Heat Cartography Map with nodes colored by their average Pearson Correlation Coefficient. Region R4 represents the switch genes. (**c**) Dendrogram and heat map for switch genes. The suffix ____D indicates the sample came from the diseased cohort. (**d**) Robustness of the correlation network.

**Figure 5:**
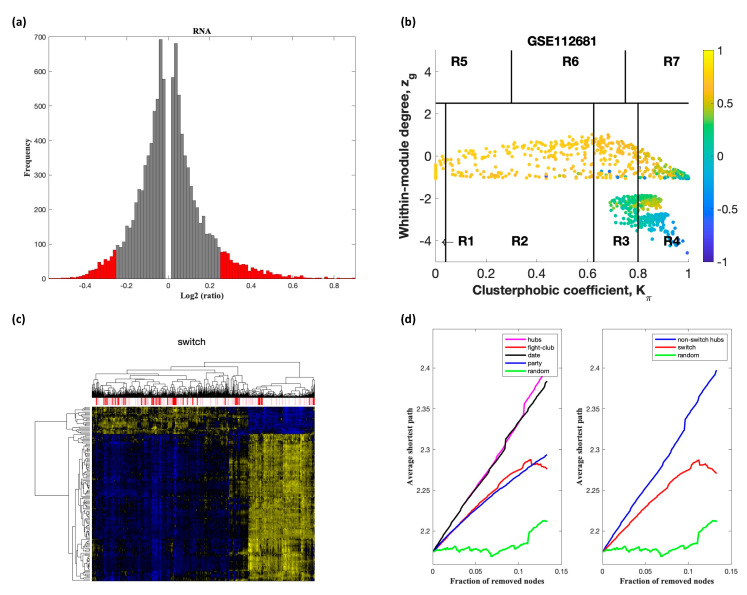
SWIM analysis of whole blood of ALS subjects in GSE112681. (**a**) Distribution of log2 fold change values where the red bars are selected for further analysis. (**b**) Heat Cartography Map with nodes colored by their average Pearson Correlation Coefficient. Region R4 represents the switch genes. (**c**) Dendrogram and heat map for switch genes. The suffix ____D indicates the sample came from the diseased cohort. (**d**) Robustness of the correlation network.

Analysis of GSE60424 containing samples from ALS and healthy controls identified nine switch genes, including *KDM6A*, *MAP7D2*, *ZFX*, *ERCC6L*, *IFI44L*, *XAF1*, *IFI44*, *EIF2S3*, and *XIST*, using a fold change cut off of 1.5 (Figure 2). Likewise, SWIM analysis of E-TABM-940 identified 100 switch genes (Figure 3). Analysis of GSE39643 containing miRNA expression data from ALS subjects and healthy controls yielded eight miRNAs, including miR-1297, miR-137, miR-193a-3p, miR-379, miR-513a-5p, miR-548g, miR-640, and miR-651, as key switch genes (Figure 4). Finally, analysis of GSE112681 yielded 133 switch genes (Figure 5). There was no overlap in switch genes among the four arrays. The list of switch genes identified in the four datasets is presented in Supplementary Table S1.

In part (a) of Figure 2, Figure 3, Figure 4 and Figure 5, the group of samples that were eliminated and retained according to the selected threshold are displayed as grey and red bars, respectively. The *x*-axis represents the fold-change value (log2 of the fold-change) that is the ratio of the average expression data in ALS patients compared to the average expression data in normal controls computed for protein-coding and non-coding RNAs. The *y*-axis represents the frequency of the obtained fold-change values.

Part (b) of Figure 2, Figure 3, Figure 4 and Figure 5 shows the correlation communities based on the average Pearson correlation coefficient. The nodes with a negative correlation value with their interaction partner, known as fight-club hubs, are depicted in R4 in blue. The plane is identified by two parameters: *Zg* (within-module degree) and *Kπ* (clusterphobic coefficient) and it is divided into seven regions each defining a specific node role (R1-R7). High *Zg* values correspond to nodes that are hubs within their module (local hubs), whereas low *Zg* values correspond to nodes with few connections within their module (non-hubs within their communities, but they could be hubs in the network). Each node is colored according to its average Pearson correlation coefficient (APCC) value. Yellow nodes are party and date hubs, which are positively correlated in expression with their interaction partners. Blue nodes are the fight-club hubs, which have an average negative correlation in expression with their interaction partners. Blue nodes falling in the region R4 are the switch genes, which are characterized by low *Zg* and by high *Kπ* values and are connected mainly outside their module.

Part (c) of Figure 2, Figure 3, Figure 4 and Figure 5 shows a dendogram and heat map of the expression of the switch genes. The expression profiles of switch genes (including protein-coding and non-coding RNAs) are clustered according to rows (switch genes) and columns (samples) of the switch genes expression data (biclustering). The colors represent different expression levels that increase from blue to yellow. The red, pink, and white bars at the top are an alternate marker of the cohorts. When the sample size is large the *x* axis labels are disabled. Red and pink denote ALS samples.

The data presented in part (d) of Figure 2, Figure 3, Figure 4 and Figure 5, show that the fight-club hubs differ from date and party hubs and switch genes are significantly different than random, thereby confirming the robustness of the analysis. The *x*-axis represents the cumulative fraction of removed nodes, while the *y*-axis represents the average shortest path. The shortest path between two nodes is the minimum number of consecutive edges connecting them. Each curve corresponds to the variation of the average shortest path of the correlation network as function of the removal of nodes specified by the colors of each curve.

### 2.3. Network and Pathway Analysis of Switch Genes in ALS

**Figure 6:**
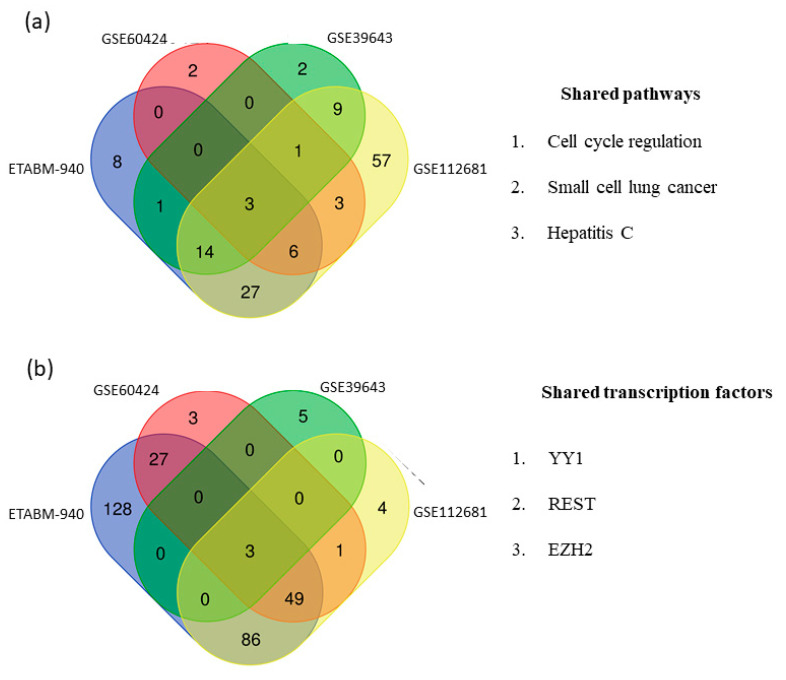
Pathway and transcription factors analyses. Pathway and transcription factors analyses were performed using NetworkAnalyst and miRNet. (**a**) Venn diagram analysis showed three shared pathways between the datasets. Results were derived from the Kyoto Encyclopedia of Genes and Genome (KEGG). (**b**) Venn diagram analysis showed three shared transcription factors between the datasets. Results were derived from the ENCODE database.

To identify the functional role and biological processes associated with the identified switch genes, we performed a network and pathway analysis using NetworkAnalyst and miRNet, [17,18]. Switch genes obtained from the four arrays were imported into miRNet and NetworkAnalyst and analyzed separately. Venn diagram analysis showed three common pathways among the datasets, including cell cycle regulation, small cell lung cancer, and hepatitis C (Figure 6a). The complete list of overrepresented pathways in each study is provided in Supplementary Table S2. We next performed a transcription factor analysis using NetworkAnalyst and miRNet [17,18] with the ENCODE database. Three transcription factors regulating the switch genes, including *REST*, *YY1,* and *EZH2*, were shared between the datasets (Figure 6b). The list of transcription factors identified in each dataset is presented in Supplementary Table S3.

### 2.4. Sex-Specific Gene Expression in the Blood of ALS Patients

After a careful inspection, we noticed that some of the switch genes, including *KDM6A* and *XIST,* have been extensively investigated in sex-specific traits due to their involvement in the process of X chromosome inactivation [19,20,21,22]. This finding prompted us to explore whether the switch genes identified in our analyses exhibited gene expression differences between males and females with ALS. We used the array E-TABM-940 that contained samples from 29 males and 28 females with ALS and 28 healthy controls for this analysis. Eight out of the nine switch genes identified in the study GSE60424 showed sex-specific differential gene expression. Six genes, including *XIST*, *KDM6A*, *ZFX*, *MAP7D2*, *EIF2S3,* and *ERCC6L,* were downregulated in males compared to females with ALS (Table 2). Furthermore, six genes, including *XIST*, *KDM6A*, *ZFX*, *XAF1*, *MAP7D2*, and *IFI44L,* were upregulated in females with ALS compared to healthy controls.

### 2.5. Identification of Sex-Associated Switch Genes, Biological Pathways, and Transcription Factors

To investigate sex-specific differences in switch genes, we reanalyzed the datasets E-TABM940 and GSE112681 by sex. These datasets contained enough samples from males and females that would provide us with enough power to determine sex differences in gene expression and biological pathways in ALS. SWIM analysis of E-TABM940 identified 14 and 115 switch genes in males and females with ALS, respectively (Supplementary Figures S2–S5). Similarly, analysis of GSE112681 identified 163 and 86 switch genes in males and females, respectively. There was no overlap in switch genes between the male datasets. One switch gene, CHD4, overlapped in the datasets from females. The complete list of switch genes is presented in Supplementary Table S4.

**Figure 7:**
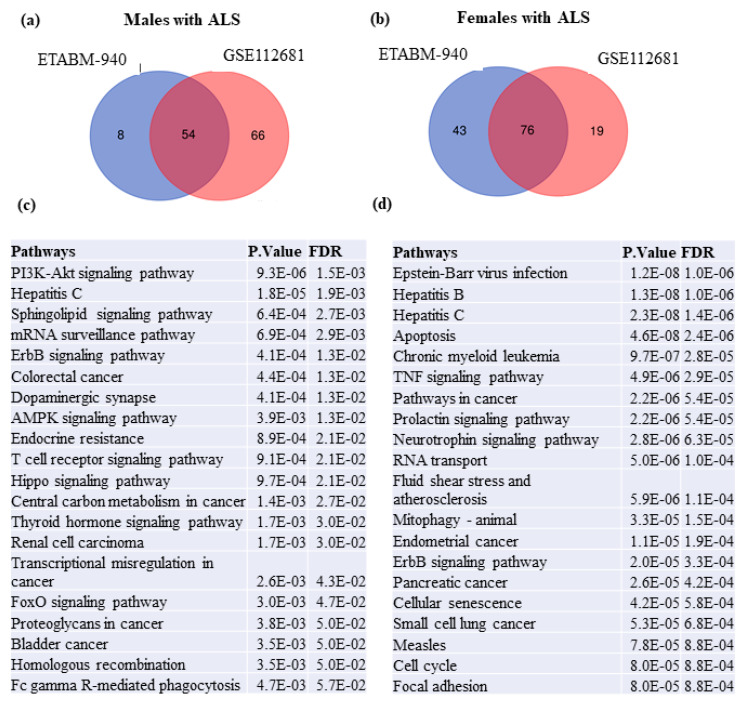
Biological and functional analysis of switch genes by sex. Biological and functional analysis was performed using NetworkAnalyst. Venn diagram analysis of biological pathways identified in switch genes from males (**a**) and females (**b**) in datasets E-TABM-990 and GSE112681. Top 20 most significant dysregulated pathways in males (**c**) and females (**d**).

Biological and functional analysis was performed for each set of switch genes from males and females independently. Network analysis of switch genes identified in males in the dataset ETABM940 associated with sphingolipid, PI3K-AKT, AMPK, FOXO signaling pathways, hepatitis C, endocrine resistance, and cancer. Likewise, switch genes from males in the dataset GSE112681 were enriched in viral carcinogenesis, Epstein–Barr virus infection, proteasome, spliceosome, alcoholism, cancer, ubiquitin-mediated proteolysis, thyroid hormone signaling, and others. Venn diagram analysis showed that switch genes from males in the datasets E-TABM940 and GSE112681 were involved in 54 shared dysregulated pathways (Figure 7a). Notably, the most significant dysregulated pathways in males were PI3K-AKT, hepatitis C, ErbB signaling, colorectal cancer, and sphingolipid signaling (Figure 7c). Metabolic and energetic pathways, including PI3K-AKT, sphingolipid, central carbon metabolism, FOXO, and AMPK, are predominant in males with ALS. The top 20 most significant dysregulated pathways in males with ALS are presented in Figure 7c.

The same analysis was performed for the switch genes obtained from the female datasets. Network analysis of female switch genes identified in the dataset E-TABM-940 associated with viral carcinogenesis, cell cycle, cancer, hepatitis B, transcriptional misregulation in cancer, ubiquitin-mediated proteolysis, pancreatic cancer, and others. Similarly, network analysis of switch genes from females in dataset GSE112681 was enriched in ribosome biogenesis, TNF signaling, hepatitis B and C, apoptosis, protein processing in the endoplasmic reticulum, IL-17 signaling, and others. Venn diagram analysis showed that switch genes identified in females in datasets E-TABM-940 and GSE112681 were enriched in 76 shared dysregulated pathways (Figure 7b). Epstein–Barr virus infection, hepatitis B and C, apoptosis, chronic myeloid leukemia, and cancer were the most significant dysregulated pathways in females. The top 20 most significant dysregulated pathways in females with ALS are presented in Figure 7d.

Transcription factor analysis was performed using the ENCODE database in NetworkAnalyst. We identified 9 and 134 transcription factors regulating the switch genes in males from datasets ETABM940 and GSE112681, respectively. Eight transcription factors were shared between these datasets (Supplementary Figure S1a). Kruppel like factor 9 (KLF9) was among the top-ranked transcription factors according to network topology measures, degree, and betweenness centrality.

Similarly, we identified 143 and 42 transcription factors regulating the switch genes in females from datasets E-TABM 940 and GSE112681, respectively. Thirty-one transcription factors were shared between the female datasets (Supplementary Figure S1b). GTF2E2, SMAD5, and TFDP1 were among the top-ranked transcription regulators of switch genes obtained from females. The nuclear factor related to kappaB binding protein (NFRKB) was the only transcription factor shared between males and females (Supplementary Figure S1c). The complete list of transcription factors is provided in Supplementary Table S5.

### 2.6. Protein–Chemical Interaction Analysis

In order to investigate potential chemicals that could be useful for treating patients with ALS, we performed a protein-chemical network analysis in NetworkAnalyst. Because males and females may respond to chemicals differently, we performed the analysis by sex. Switch genes from males and females corresponding to E-TABM940 and GSE112681 datasets were analyzed separately. Chemicals were ranked according to network topology measurements, degree, and betweenness centrality. Valproic acid and cyclosporine were the highest ranked chemicals in both males and females (Supplementary Table S6).

## 3. Discussion

### 3.1. Identification of Switch Genes in ALS

This study used co-expression networks implemented by the SWIM software to investigate the molecular underpinnings in ALS. Using this approach, we identified several switch genes associated with the transition from a healthy state to ALS patients. SWIM analysis successfully identified 117 switch genes in four independent microarrays containing blood transcriptomic data from ALS patients and healthy controls. However, there was no overlap in switch genes between the studies. This finding may be explained by the differences in the array platforms and methods. For instance, GSE60424 used gene expression profiling by high throughput RNA sequencing, a more robust gene expression study, whereas GSE39643 used noncoding miRNA profiling, and E-TABM-940 was conducted using standard microarrays.

Several of the switch genes identified have been implicated in processes related to neurodegeneration. For example, *XAF1* encodes a protein that suppresses the activity of the inhibitor of apoptosis proteins (IAP) [23] and may play a role in the cell death of injured motoneurons [24]. Depletion of *MAP7D2* is associated with defects in axonal development and neuronal migration [25]. Mutations in *EIF2S3* have been linked to MEHMO syndrome development, an X-linked intellectual disability disorder [26].

Interestingly, three switch genes, *XIST*, *ZFX*, and *KDM6A,* have been associated with X chromosome inactivation, the process by which mammals compensate for the unequal number of sex chromosomes. *XIST* is an RNA gene located in the X chromosome inactivation center (XIC) essential for the X chromosome inactivation [27]. In the context of neurodegeneration, dysregulation of *XIST* and X chromosome inactivation has been reported in several Alzheimer’s disease (AD) studies [27]. For example, *XIST* is upregulated in brain samples from AD patients compared to controls [28]. Knockdown of *XIST* reduced Aβ_25-35_ induced toxicity, oxidative stress, and apoptosis in primary cultured rat hippocampal neurons by targeting miR-132 [28]. Similarly, silencing of *XIST* attenuated neurotoxic effects and Aβ_1-42_ expression in a rodent model of AD through miR-124 [29], suggesting its potential as a therapeutic target for AD. Nevertheless, similar studies investigating the process of X chromosome inactivation and its implications in sex-specific differences have not been documented in ALS.

Likewise, *KDM6A*, an X chromosome encoded demethylase belongs to the small subset of X-linked genes that escape X chromosome inactivation in mice and humans [30]. Strikingly, a recent study revealed that a second X chromosome conferred resilience to AD-related vulnerability through Kdm6a in mice [31]. Furthermore, *KDM6A* mRNA expression was significantly upregulated in the brains of both females with and without AD compared to males. The same study found that a genetic variant in *KDM6A* was associated with a lesser degree of cognitive decline in humans [31]. Furthermore, Kdm6a promoted the transcription of proinflammatory cytokines IL6 and IFN-β through an epigenetic mechanism revealing a potential role in innate immunity [32].

Another switch gene identified in the blood of ALS subjects, *ZFX*, is an X chromosome-linked gene encoding a zinc finger protein that escapes X chromosome inactivation in humans [33]. *ZFX* regulates the expression of SET nuclear proto-oncogene (*SET*), also known as protein phosphatase 2A inhibitor (*I2PP2A*) [34], a gene associated with tau protein hyperphosphorylation in Alzheimer’s disease [35]. Similar to AD, the identification of switch genes like *XIST*, *KDM6A,* and *ZFX,* is intriguing and may indicate that the process of X chromosome inactivation may be altered in ALS patients. Furthermore, the process of X chromosome inactivation and dysregulation in these genes may explain the higher prevalence of neurodegenerative diseases in males than females.

### 3.2. Sex-Specific Gene Expression Patterns in ALS Switch Genes

Because of the intrinsic association of several switch genes with the process of X chromosome inactivation, we next explored whether the expression of switch genes was altered by sex in the blood of individuals with ALS. Eight switch genes, including *XIST*, *KDM6A*, *ZFX*, *MAP7D2*, *XAF1*, *EIF2S3*, and *IFI44L*, exhibited sex-specific gene expression changes in the blood of ALS subjects. The sex-specific gene expression patterns observed in these genes, particularly in those genes involved in X chromosome inactivation, may provide a molecular rationale for the higher prevalence of ALS among males. In this context, the vast majority of epidemiological studies indicate that males are at higher risk for ALS [6,7]. Indeed, these studies have established that male sex is a well-documented risk factor for ALS, with a male to female ratio of 1.2–1.5 [3]. Interestingly, sex differences in ALS patients are not observed in postmenopausal women suggesting that sex hormones may also be significant risk factors [36]. Sex-specific differences are not only observed in sporadic cases. For example, a meta-analysis including 12,784 subjects reported a higher prevalence of females *C9orf72* carriers with ALS than males, suggesting that sex-specific factors may be responsible for the pathogenic mechanisms C9orf72 related ALS [37]. In addition, C3orf72, a risk factor for ALS, was significantly increased in different brain regions of male patients with ALS compared to females [38].

### 3.3. Biological and Functional Analyses of Switch Genes

Biological and functional analyses of switch genes revealed three shared pathways across the studies, including cell cycle regulation, hepatitis C, and small cell lung cancer. In this regard, very few studies have reported a possible link between ALS and hepatitis C. One study identified a complex formed by a structural domain in the hepatitis C virus and the *VAP*-*MSP* domain that contains ALS causing mutations [39], suggesting a potential link between both diseases [40]. In addition, a case report presented evidence of ALS symptoms in an individual with a nine-year history of hepatitis C [41]. Nevertheless, the evidence is minimal, and more studies are needed to investigate a potential link between ALS and hepatitis C.

Several studies have indicated that disturbances in the cell cycle program may be associated with neurodegeneration and ALS. For example, one study reported the upregulation of cell cycle-related miRNAs in a genetic model of ALS G93A-SOD1 mice [42]. Cell division cycle kinase 7 (*CDC7*) plays a role in the hyperphosphorylation of TDP-43, a central protein in the pathogenesis of ALS [43]. Notably, *CDC7* inhibitors have shown promise in ALS animal and cellular models by reducing TDP43 phosphorylation [44,45]. Furthermore, a small molecule inhibitor of *CDC7* reduced TDP-43 phosphorylation and prevented neurodegeneration in TDP-43-transgenic animals [43]. These results suggest that cell cycle inhibitors of *CDC7* may be potential therapeutics for TDP-43 associated proteinopathies, including ALS. Further studies investigating the importance of cell cycle regulation in ALS are warranted.

Transcription factor analysis of switch genes identified three transcription regulators, *REST*, *YYI*, and *EZH2*, shared between the three datasets. Among these transcription factors, *REST* plays a role in neuron development, normal aging, neuroprotection, and neurodegeneration. *REST,* also known as neuron restrictive silencing factor (*NRSF*), represses neuronal gene transcription in non-neuronal cells, and it is downregulated upon completion of neuronal cell differentiation [46]. Interestingly, *REST* regulates a genetic network that mediates cell death, stress resistance, and neurodegeneration [47]. The same study showed that once this genetic network is dysregulated, REST is lost from the nucleus and presents in autophagosomes with pathological misfolded proteins in AD, frontotemporal dementia, and dementia with Lewy bodies [47]. Furthermore, *REST* expression is associated with the preservation of cognitive function and longevity [47]. Moreover, chronic exposure to polychlorinated biphenyls (PCBs), environmental toxins associated with neurodegeneration, enhanced *REST* mRNA and protein expression levels in cellular models, and REST downregulation via small interference RNA prevented PCBs-mediated cell death [48]. Collectively, these studies suggest that targeting *REST* may be a possible therapeutic route for neurodegenerative diseases, including ALS.

### 3.4. Sex-Associated Switch Genes, Biological Pathways, and Transcription Factors

To investigate sex-specific differences in switch genes in ALS, we reanalyzed the datasets E-TABM940 and GSE112681 by sex. We identified switch genes associated with drastic gene expression changes in males and females with ALS. There was no overlap between switch genes from males and females. Biological and functional analyses of switch genes from male and female datasets identified several differences in biological pathways. Switch genes identified in males were predominantly enriched in the PI3K-AKT, hepatitis C, ErbB, colorectal cancer, and sphingolipid signaling pathways. The most significant dysregulated pathway in males was the PI3K-AKT pathway. In this context, a network analysis study identified the PI3K-AKT signaling pathway as the most significantly dysregulated pathway in ALS patients [49]. Several other studies have identified the PI3K-AKT as an important molecular pathway in the pathogenesis of ALS. For example, in vivo and in vitro studies in a genetic mice model of ALS suggested that PI3K-AKT is inhibited and is responsible for the reduced viability of motor neurons [50]. Furthermore, endothelin-1, a vasoactive peptide produced by activated astrocytes and microglia, is associated with motor neuron cell death in ALS [51]. The associated neurotoxicity of endothelin 1 may be mediated by inhibition of the PI3K-AKT pathway [52]. Although there is no direct evidence for the involvement of the PI3K-AKT pathway specifically in males with ALS, its potential involvement in the pathogenesis of ALS in male subjects warrants further investigation.

Switch genes identified in females associated with Epstein–Barr virus infection, hepatitis B and C, apoptosis, and chronic myeloid leukemia. The most significant dysregulated pathway in females with ALS was the Epstein–Barr virus infection. EBV infection is linked to multiple sclerosis in women, but the mechanism underlying this association is mostly unclear [53]. Notably, switch genes obtained from females are enriched in infectious diseases, inflammatory pathways, apoptosis, and atherosclerosis. In contrast, metabolic and energetic pathways, including PI3K-AKT, sphingolipid, central carbon metabolism, FOXO, and AMPK signaling pathways, were more represented in males. In this regard, PI3K-AKT, FOXO, and AMPK play prominent roles in insulin signaling and glucose metabolism [54]. This finding is notable considering the numerous studies suggesting the impairment in insulin signaling and glucose metabolism as the culprit for neurodegenerative diseases [55]. These results suggest that different molecular pathways may be involved in the pathogenesis of ALS in males and females.

In addition, we also observed some differences in transcription factor regulators between males and females. Network analysis identified KLF9 as a highly ranked transcription regulator of switch genes in the male datasets. Interestingly, KLF9 has been suggested to play a role in regulating hepatic glucose metabolism and glucocorticoid-induced diabetes [56,57]. Moreover, KLF9 has been implicated in axonal regeneration and growth [58]. Thus, KLF9 may be explored as a potential therapeutic target in ALS.

Similarly, transcription factor analysis identified GTF2E2, SMAD5, and TFDP1 as highly ranked transcription factors in the female datasets. GTF2F2 has been associated with cancer, human immunodeficiency virus (HIV-1) replication, and DNA repair pathways [59,60,61]. Similarly, SMAD5 has been widely implicated in apoptosis, cancer, and the development of the nervous system [62,63,64,65]. There is no evidence for the involvement of these transcription factors with neurodegeneration or with sex-specific pathways. TFDP1, which encodes a protein that controls the G1/S phase of the cell cycle, is downregulated in women with endometriosis [65]. Likewise, there is no direct association between TFDP1 and neurodegeneration. NFRKB was the only transcription factor shared between males and females. This finding is not unexpected since NFRKB plays a pivotal role in inflammation, a common process involved in neurodegenerative diseases’ pathogenesis [66,67].

With regards to inflammation, one hypothesis suggests that sex-specific factors in the CNS immune system may contribute to the observed pathological and clinical differences between males and females in neurodegenerative diseases [68]. The authors hypothesize that sex-specific microglial regulation of the type I interferon response and STAT3 signaling may explain some of the sex differences in ALS patients [68]. For example, loss of C9orf72 triggered a hyperactive type I interferon response with enhanced autoimmunity and anti-tumor immunity in mice [69]. This increased interferon production was also present in whole blood and brain samples from C9orf72 ALS patients [69]. Furthermore, the proinflammatory transcription factor STAT3 is activated in the spinal cord microglia of ALS patients, and it is increased in interferon induced-cell types in female mice compared to male [70,71]. These findings suggest that a hyperactive type I interferon response and STAT3 activation may confer a stronger immune system in females and consequently, a higher risk of ALS in males. Additional biochemical and functional studies are necessary to understand the association between these transcription factors in ALS.

### 3.5. Protein–Chemical Network Analysis Identifies Potential Therapeutics for ALS

Valproic acid and cyclosporine were identified as the highest ranked chemicals interacting with switch genes from both males and females with ALS. Several studies have identified valproic acid as a potential drug for neurodegenerative diseases including Alzheimer and Parkinson’s diseases. For example, valproic acid rescued the neuronal loss in the brain of APP/PS1 double transgenic AD mice model [72]. Interestingly, valproic acid elicited greater neuroprotective effects in the male AD mice compared to female [73]. Valproic acid reduced synaptic damage in Parkinson and prion diseases via inhibition of cytoplasmic phospholipase A2 (cPLA2) signaling [74]. More recently, the combination of valproic acid and estrogen enhanced sensitivity to estrogen therapy, reduced amyloid beta aggregation, and significantly improve cognitive functions in ovariectomized mice with AD suggesting it could be a potential therapeutic for postmenopausal women with AD [75]. In addition, a bioinformatic study revealed that valproic acid may be neuroprotective for Alzheimer’s disease, vascular dementia, and frontotemporal dementia [15].

Cyclosporine is an immunosuppressant commonly used to prevent organ transplant rejection and rheumatoid arthritis. Like valproic acid, cyclosporine has been shown to provide neuroprotective effects in neurodegenerative diseases. For instance, cyclosporine alleviated the aggregation of alpha synuclein, mitochondrial dysfunction, and dopaminergic cell death in a rat model of cypermethrin-induced Parkinsonism [76]. Furthermore, cyclosporine improved synaptic and non-synaptic mitochondrial respiration after traumatic brain injury in rats [77]. Future preclinical studies testing these drugs in cellular and animal models of ALS is warranted.

### 3.6. Conclusions

Sex differences may have profound effects on disease susceptibility, pathophysiology, and progression. Understanding sex-specific differences are crucial for patient treatment. Women with ALS are likely to respond differently to treatment than men. In this regard, several studies indicate that women are not only less susceptible to ALS but also present with less severe symptoms and slower progression [6,7]. Here we identified a set of genes that exhibited differences in gene expression between males and females. Notably, some of the switch genes have been implicated in X chromosome inactivation suggesting this process may be involved in ALS. These findings may explain the evidence from epidemiological studies indicating a higher prevalence of ALS among males. Furthermore, we identified sex differences in biological pathways in ALS patients. For example, biological pathways disrupted in males with ALS were predominantly associated with metabolic and energetic pathways, whereas pathways related to infection, inflammation, and apoptosis were more predominant in females with ALS. Protein–drug interaction analysis indicated that valproic acid and cyclosporine are potential drugs for the treatment of ALS. In addition, the switch genes identified in this study may be useful biomarkers in studies investigating sex-specific differences in ALS patients. Future studies investigating these genes’ potential functional role in driving disease sex disparities in ALS are warranted.

## 4. Methods

### 4.1. Database Mining

The terms “Amyotrophic lateral sclerosis”, “Lou Gehrig’s Disease”, “blood”, “human”, “RNA”, and “microarray” were used to search the NCBI GEO database (https://www.ncbi.nlm.nih.gov/gds) and ArrayExpress database (https://www.ebi.ac.uk/arrayexpress/) on 1 September 2020. We identified seven transcriptomic arrays from individuals with ALS. Only studies with at least five samples from individuals diagnosed with ALS and controls were analyzed further.

### 4.2. Clinical and Demographic Characteristics of Participants Analyzed in the Study

The clinical information about the study participants for the dataset GSE39643 has been published elsewhere [78]. Briefly, blood was collected from participants recruited from the Massachusetts General Hospital (MHG) and MGH Neurology Clinical Trials Unit. The study included 8 sporadic ALS patients and 8 healthy controls. Subjects receiving an experimental drug or with unstable medical conditions unrelated to ALS were excluded. The average age of patients was 58.8 ± 10.8, and 59% were males. The revised ALS Functional Rating scale (ALSFRS-R) was used to assess disease severity. The dataset E-TABM-940 included 56 sporadic ALS and 28 healthy controls. The average age of ALS patients was 57.8 ± 12.7. Blood samples were collected from the Methodist Hospital Research Institute, Houston, Texas, University of California, Irvine, and Mount Sinai Medical Center, New York. The ALFRS was used to assess disease severity. More detailed information about the study participants has been published in [79]. The dataset GSE112681 included 233 ALS patients recruited from the University Medical Center Utrecht, The Netherlands. Healthy controls were population-based individuals matched for gender, age, and free of neuromuscular disease. This dataset included 143 and 90 samples from males and females with ALS, respectively. The mean age of ALS patients and controls was 64.7 and 62.9 years, respectively. Patients with primary lateral sclerosis and progressive muscular atrophy were excluded from the study. The clinical information for the subjects included in the study GSE60424 is not available. Informed consent was obtained from the study participants, and the relevant ethical committees approved study protocols at each clinical site.

### 4.3. Identification of Switch Genes by SWIM Analysis

The SWItchMiner (SWIM) software was used to identify switch genes using datasets from the ALS subjects and healthy controls as previously described [14,15]. Using this software, co-expression networks are built using the Pearson correlation coefficient between two genes’ expression. The nodes of the network are RNA transcripts, and the connections between nodes represent either a significant correlation or anti-correlation of the expression of the genes. The algorithm identifies communities in the network using the k-means clustering algorithm, employing the sum of squared errors (SSE) values to determine the appropriate number of clusters. A heat cartography map of the nodes is created according to their topological properties Extracted switch genes mark the transition from the healthy to a disease state. Here, SWIM was employed to identify genes in the blood responsible for the transition from healthy to ALS.

The raw datasets from the studies listed in Table 1 were imported into SWIM. In the pre-processing phase, genes that are not expressed or only slightly expressed are removed. In the filtering phase, the fold-change limit was set between 1.5 and 4 to obtain between 1000 and 2000 genes for network analysis, and genes that were not significantly expressed differently between ALS patients compared to controls are removed. A False Discovery Rate (FDR) method was applied to correct for multiple tests. Pearson correlation analysis was used to build a co-expression network of genes differentially expressed between ALS patients and controls as previously described [16]. The k-means algorithm was used to identify communities within the network. To determine the number of clusters, SWIM uses Scree plot, which allows replicating the clustering many times with a new set of initial cluster centroid positions, and for each replicate, the k-means algorithm performs iterations until the minimum of the SSE function is reached. The cluster configuration with the lowest SSE values among the replicates is designated as the number of clusters. The heat cartography map is built using a clusterphobic coefficient *Kπ*, which measures external and internal node connections, and the global within-module degree *Zg*, which measures the extent each node is connected to others in its community. A node is considered a hub when *Zg* exceeds 5. The average Pearson correlation coefficient (APCC) between the expression profiles of each node and its nearest neighbors is used to build the heat cartography map. Using APCC, three types of hubs may be identified. Date hubs show low positive co-expression with their partners (low APCC), party hubs show high positive co-expression (high APCC), and nodes that have negative APCC values are called fight-club hubs [16]. In the final step of SWIM analysis, switch genes are identified that are a subset of the fight-club hubs that interact outside of their community. Switch genes are characterized as not being a hub in their cluster (low *Zg* < 2.5), having many links outside their cluster (*Kπ* > 0.8, when *Kπ* is close to 1 most of its links are external to its module), and having a negative average weight of incident links (APCC < 0) [16].

### 4.4. Identification and Analysis of Switch Genes by Sex

Datasets ETABM-940 and GSE112681 containing samples from male and female patients with ALS were analyzed by sex. Switch genes were identified using the SWIM software as described in previous steps. For the SWIM analysis of dataset ETABM940, we used a fold change cut-off of 1.5 for males and 2.5 for females. For the SWIM analysis of GSE261181 we used a fold change of 1.2. Switch genes obtained from males and females were analyzed independently for pathways and transcription factor analyses using NetworkAnalyst as described in previous steps.

### 4.5. Pathway and Transcription Factor Analyses

Official gene symbols for the switch genes were imported into NetworkAnalyst and miRNet, https://www.networkanalyst.ca/, https://www.mirnet.ca/miRNet/home.xhtml (accessed on 21 February 2021). The minimum connected network was selected for further pathway analysis. Data derived from the Kyoto Encyclopedia of Genes and Genome (KEGG) was used for biological pathway analysis.

The identification of transcription factors that potentially regulate the switch genes was done using NetworkAnalyst and miRNet. Transcription factor data was derived from the Encyclopedia of DNA Elements (ENCODE) ChIP-seq data. ENCODE uses the BETA Minus Algorithm in which only peak intensity signal <500 and the predicted regulatory potential score <1 is used. Shared pathways and transcription factors were identified using a Venn diagram analysis. Transcription factor analysis was ranked according to network topology measures, degree, and betweenness centrality.

### 4.6. Genes–Chemical Interaction Analysis

Switch genes from males and females were imported into NetworkAnalyst for protein–chemical interaction analysis. The chemical–gene interaction data available for analysis in NetworkAnalyst is derived from the Comparative Toxicogenomics database (CTD) from November 2016. Chemicals were ranked according to the degree and betweeness centrality.

## Figures and Tables

**Table 1:** Gene expression studies analyzed by the Switch Miner software.

Dataset	Tissue/Cells	Description of Cases and Controls	Platform
GSE39643	Blood monocytes	8 sporadic ALS, 8 healthy controls	NanoString nCounter Human miRNA assay
GSE60424	Whole blood	6 sporadic ALS	Illumina HiScanSQ
E-TABM-940	Whole blood	56 sporadic ALS, 28 healthy controls	Affymetrix GeneChip Human Genome U133 2.0
GSE28253	Blood lymphocytes	11 sporadic ALS, 11 healthy controls	Agilent-014850 Whole Human Genome Microarray
GSE112681 GSE112676 GSE112680	Whole blood	233 sporadic ALS, 508 healthy controls	Illumina HumanHT-12 v4.0

**Table 2:** Sex-specific differences in switch genes identified in the blood of ALS patients. Differential gene expression results were obtained from the Base Space Correlation Engine database. The array analyzed E-TABM-940 contained samples from 29 males, 28 females, and 28 healthy controls. FC indicates fold change.

Switch gene	Males vs. Healthy	Females vs. Healthy	Males vs. Females
*XIST*	Not significant	Up: FC = 3.58 (*p* = 9.7 × 10^−7^)	Down: FC = −489 (*p* = 1.8 × 10^−31^)
*KDM6A*	Not significant	Up: FC = 2.08 (*p* = 3.9 × 10^−5^)	Down: FC = −1.57 (*p* = 3.5 × 10^−10^)
*ZFX*	Not significant	Up: FC = 2.4 (*p* = 7.5 × 10^−9^)	Down: FC = −1.68 (*p* = 7.4 × 10^−9^)
*XAF1*	Not significant	Up: FC = 1.75 (*p* = 7.5 × 10^−7^)	Not significant
*MAP7D2*	Not significant	Up: FC = 1.59 (*p* = 1.7 × 10^−3^)	Down: FC= –3.80 (*p* = 2.0 × 10^−17^)
*EIF2S3*	Not significant	Not significant	Down: FC= −1.37 (*p* = 1.0 × 10^−4^)
*IFI44L*	Not significant	Up: FC = 2.24 (*p* = 3.0 × 10^−4^)	Not significant
*ERCC6L*	Not significant	Not significant	Down: FC = −1.24 (*p* = 6.0 × 10^−4^)

## Data Availability

Not applicable.

## Supplementary Materials

The following are available online at https://www.mdpi.com/article/10.3390/ijms22137150/s1.
