# Supplementary material for: Network Analysis Identifies Sex-Specific Gene Expression Changes in Blood of Amyotrophic Lateral Sclerosis Patients
Source: Int J Mol Sci. 2021 Jul 1;22(13):7150. doi: 10.3390/ijms22137150 (PMC8269377; doi:10.3390/ijms22137150)
Supplement: Supplementary file 1 [file ijms-22-07150-s001.zip › ijms-1274696-supplementary-revised20210611.pdf]

Supplemental information

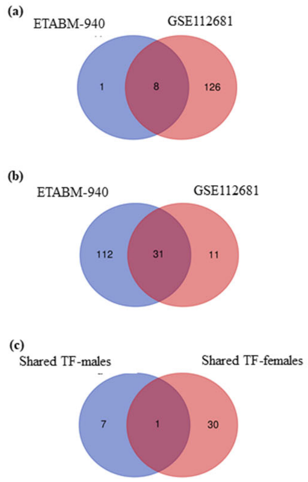

Supplementary figure S1. Transcription factor analysis by sex. Transcription factor analysis was performed using NetworkAnalyst. ENCODE database was selected for the analysis. (a) Venn diagram analysis showed that 8 transcription factors were shared in males from datasets ETABM940 and GSE112681. (b) Thirty-one transcription factors were shared in females from the same datasets. (c) One transcription factor was shared between males and females.

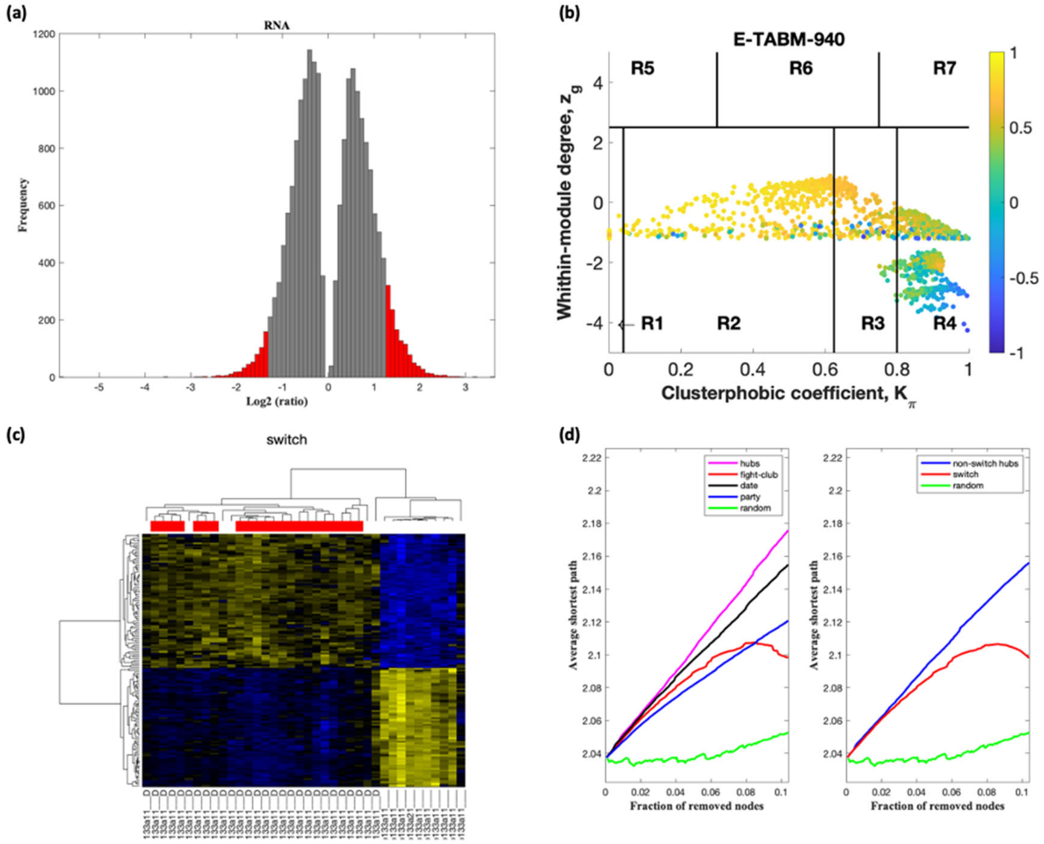

Supplementary figure S2. SWIM analysis of whole blood of female ALS subjects in E-TABM-940. **(a)** Distribution of log2 fold change values where the red bars are selected for further analysis. **(b)** Heat Cartography Map with nodes colored by their average Pearson Correlation Coefficient. Region R4 represents the switch genes. **(c)** Dendrogram and heat map for switch genes. The suffix ____D or red at the top indicate the sample came from the diseased cohort. **(d)** Robustness of the correlation network.

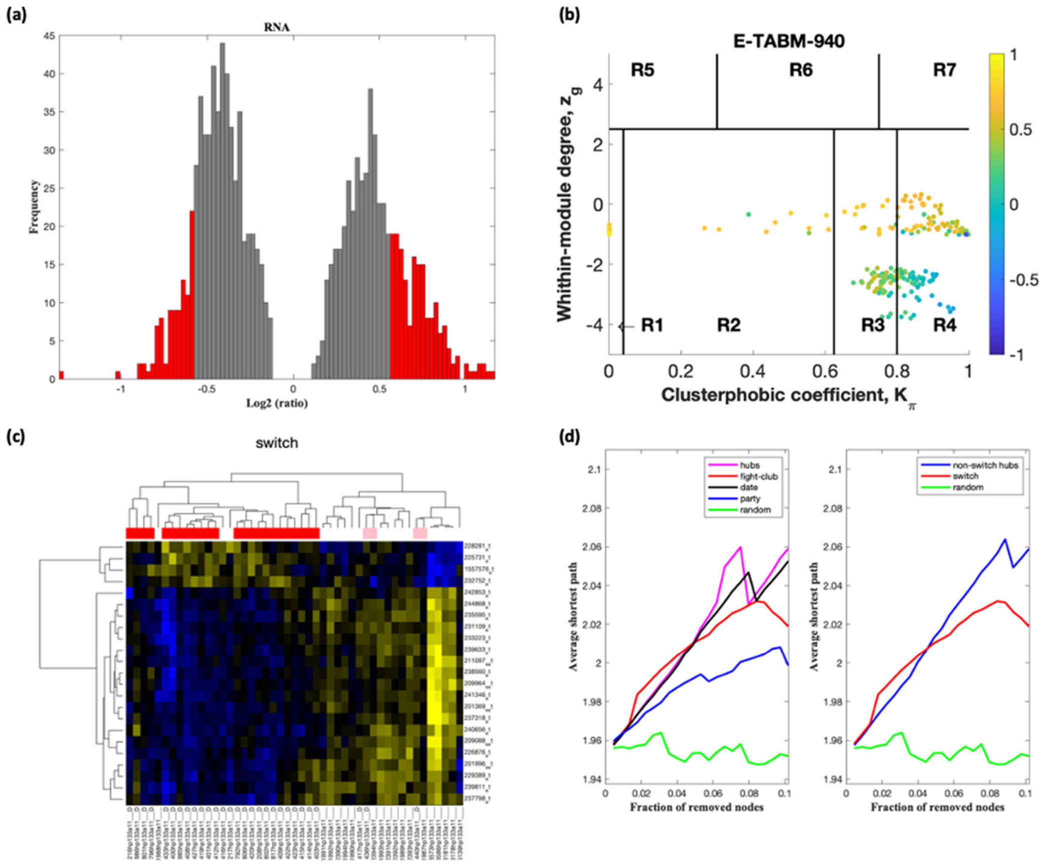

Supplementary figure S3. SWIM analysis of whole blood of male ALS subjects in E-TABM-940. **(a)** Distribution of log2 fold change values where the red bars are selected for further analysis. **(b)** Heat Cartography Map with nodes colored by their average Pearson Correlation Coefficient. Region R4 represents the switch genes. **(c)** Dendrogram and heat map for switch genes. The suffix ____D or red at the top indicate the sample came from the diseased cohort. **(d)** Robustness of the correlation network.

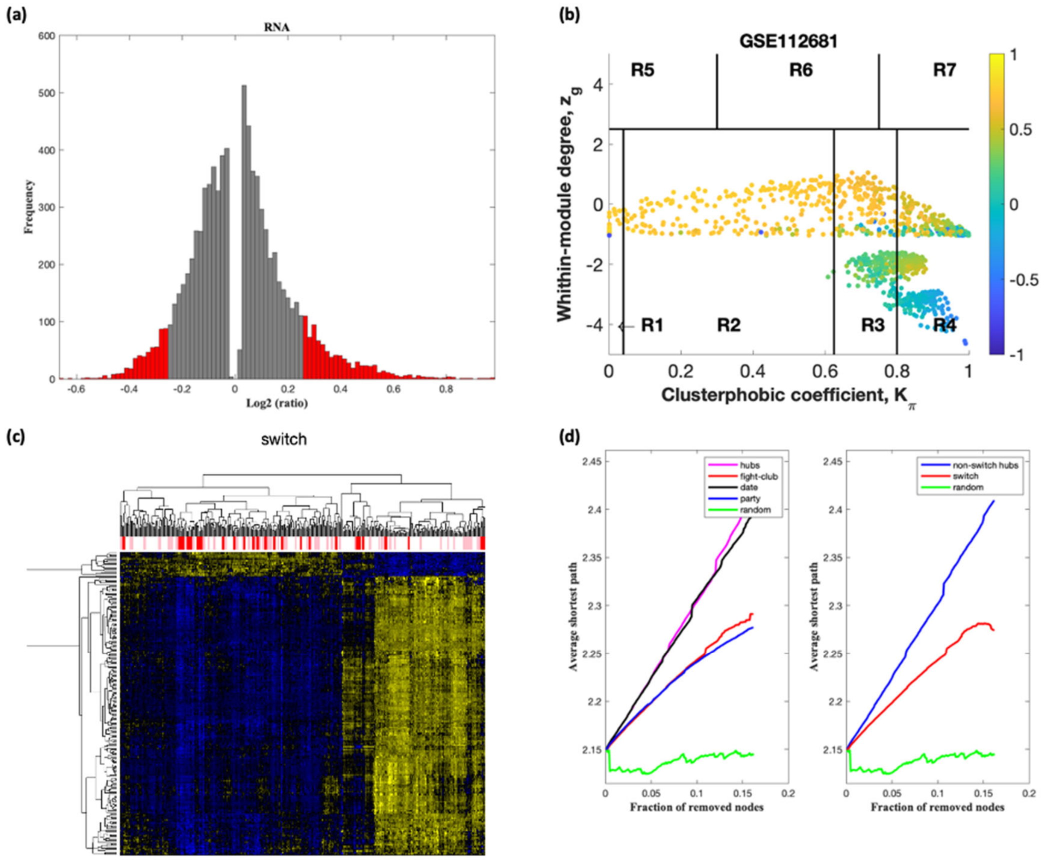

Supplementary figure S4. SWIM analysis of whole blood of male ALS subjects in GSE112681. **(a)** Distribution of log2 fold change values where the red bars are selected for further analysis. **(b)** Heat Cartography Map with nodes colored by their average Pearson Correlation Coefficient. Region R4 represents the switch genes. **(c)** Dendrogram and heat map for switch genes. Red or pink at the top indicate the sample came from the diseased cohort. **(d)** Robustness of the correlation network.

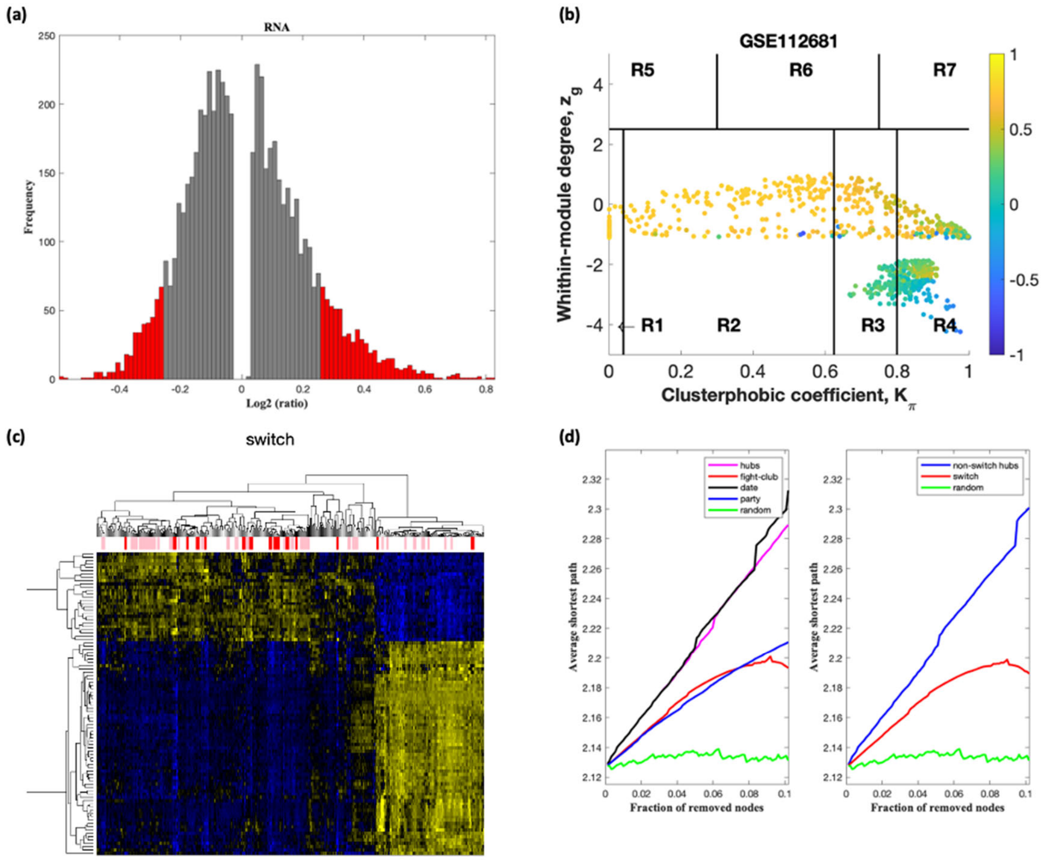

Supplementary figure S5. SWIM analysis of whole blood of female ALS subjects in GSE112681. **(a)** Distribution of log2 fold change values where the red bars are selected for further analysis. **(b)** Heat Cartography Map with nodes colored by their average Pearson Correlation Coefficient. Region R4 represents the switch genes. **(c)** Dendrogram and heat map for switch genes. Red or pink at the top indicate the sample came from the diseased cohort. **(d)** Robustness of the correlation network.

Supplementary tables

Supplementary table S1. Switch genes in blood of ALS patients.

Supplementary table S2. Pathway analysis of switch genes in blood of ALS patients compared to healthy controls.

Supplementary table S3. Transcription factor analysis of switch genes in blood of ALS patients compared to healthy controls.

Supplementary table S4. Switch genes identified in blood of males and females with ALS compared to healthy controls.

Supplementary Table S5. Transcription factor analysis of switch genes from males and females with ALS.

Supplementary Table S6. Chemical-protein analysis of switch genes identified in males and females with ALS
